# Supplementary figures and images for: TrkA-mediated endocytosis of p75-CTF prevents cholinergic neuron death upon γ-secretase inhibition
Source: Life Sci Alliance. 2021 Feb 3;4(4):e202000844. doi: 10.26508/lsa.202000844 (PMC7898468; doi:10.26508/lsa.202000844)

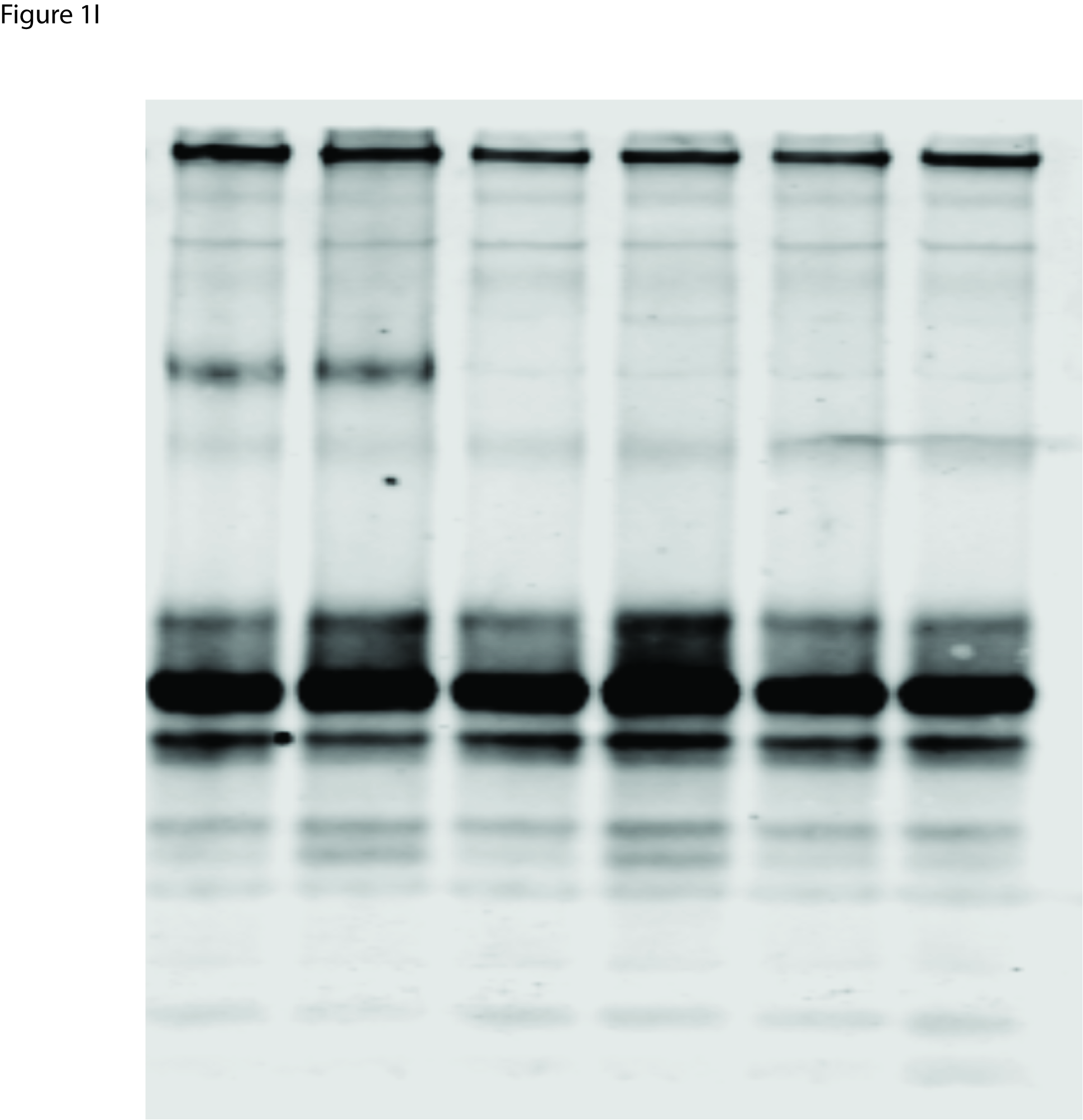

Supplement: Supplementary file 1 [file LSA-2020-00844_SdataF1.tif]

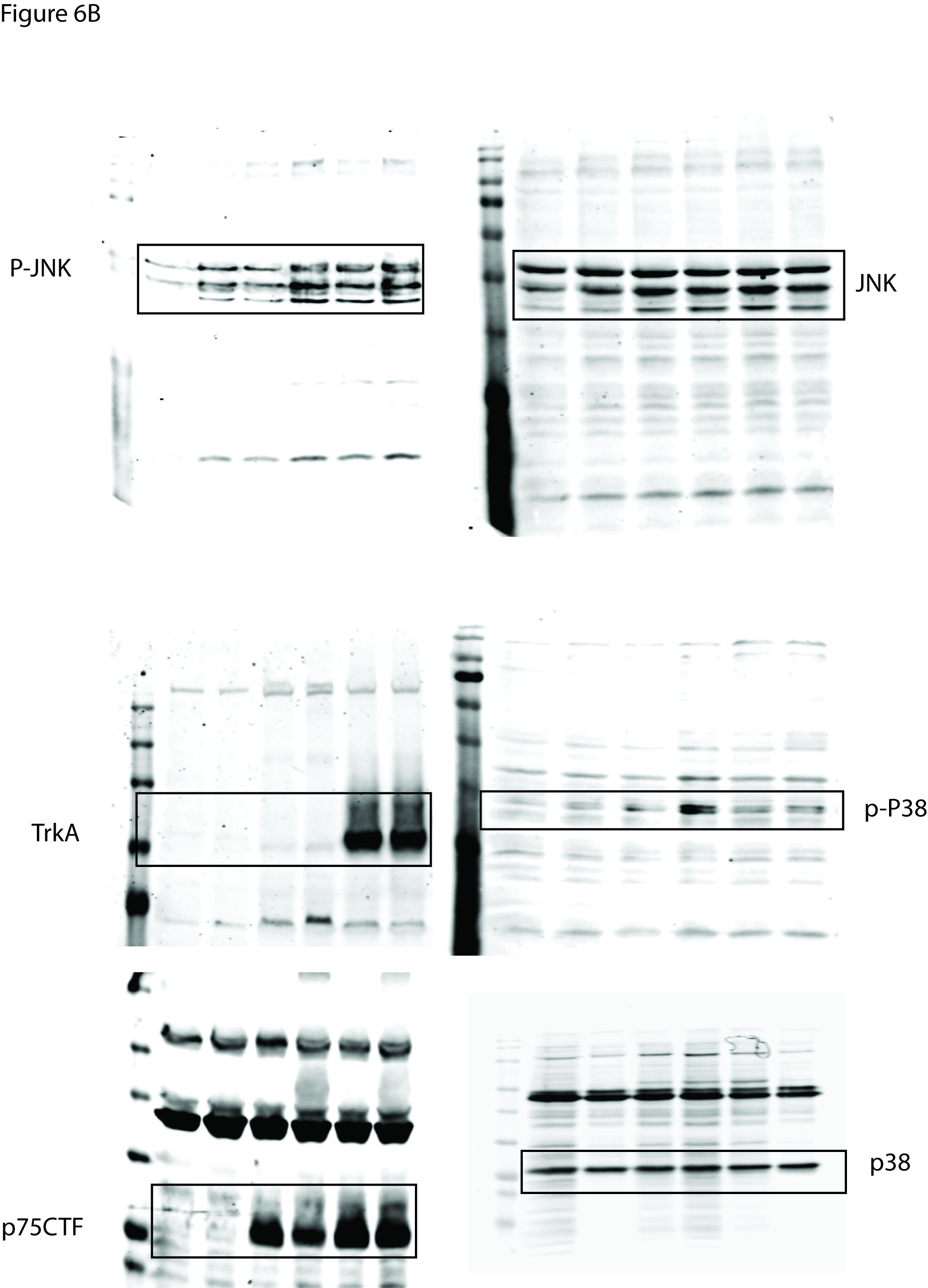

Supplement: Supplementary file 2 [file LSA-2020-00844_SdataF6.tif]
